# Epigenetic inactivation of the *NORE1 *gene correlates with malignant progression of colorectal tumors

**DOI:** 10.1186/1471-2407-10-577

**Published:** 2010-10-22

**Authors:** Chang Kyun Lee, Jin-Hee Lee, Min-Goo Lee, Seong-In Jeong, Tae-Kyu Ha, Min-Ju Kang, Byung-Kyu Ryu, Young Hwangbo, Jae-Jun Shim, Jae Young Jang, Kil Yeon Lee, Hyo Jong Kim, Sung-Gil Chi

**Affiliations:** 1 Division of Gastroenterology, Department of Internal Medicine, Kyung Hee University School of Medicine, Seoul, Korea; 2 School of Life Sciences and Biotechnology, Korea University, Seoul, Korea; 3 Department of Surgery, Kyung Hee University School of Medicine, Seoul, Korea

## Abstract

**Background:**

NORE1 (RASSF5) is a newly described member of the RASSF family with Ras effector function. *NORE1 *expression is frequently inactivated by aberrant promoter hypermethylation in many human cancers, suggesting that NORE1 might be a putative tumor suppressor. However, expression and mutation status of *NORE1 *and its implication in colorectal tumorigenesis has not been evaluated.

**Methods:**

Expression, mutation, and methylation status of *NORE1A *and *NORE1B *in 10 cancer cell lines and 80 primary tumors were characterized by quantitative PCR, SSCP, and bisulfite DNA sequencing analyses. Effect of NORE1A and NORE1B expression on tumor cell growth was evaluated using cell number counting, flow cytometry, and colony formation assays.

**Results:**

Expression of *NORE1A *and *NORE1B *transcript was easily detectable in all normal colonic epithelial tissues, but substantially decreased in 7 (70%) and 4 (40%) of 10 cancer cell lines and 31 (38.8%) and 25 (31.3%) of 80 primary carcinoma tissues, respectively. Moreover, 46 (57.6%) and 38 (47.5%) of 80 matched tissue sets exhibited tumor-specific reduction of *NORE1A *and *NORE1B*, respectively. Abnormal reduction of *NORE1 *was more commonly observed in advanced stage and high grade tumors compared to early and low grade tumors. While somatic mutations of the gene were not identified, its expression was re-activated in all low expressor cells after treatment with the demethylating agent 5-aza-dC. Bisulfite DNA sequencing analysis of 31 CpG sites within the promoter region demonstrated that abnormal reduction of *NORE1A *is tightly associated with promoter CpG sites hypermethylation. Moreover, transient expression and siRNA-mediated knockdown assays revealed that both NORE1A and NORE1B decrease cellular growth and colony forming ability of tumor cells and enhance tumor cell response to apoptotic stress.

****Conclusion**:**

Our data indicate that epigenetic inactivation of *NORE1 *due to aberrant promoter hypermethylation is a frequent event in colorectal tumorigenesis and might be implicated in the malignant progression of colorectal tumors.

## Background

Mutational activation of *Ras *plays a critical role in the development and malignant progression of many human tumors [1,2]. Ras oncoproteins participate in the regulation of a broad range of biological processes, including cell growth and differentiation, membrane trafficking, transcriptional regulation and apoptosis [2,3]. Ras proteins exist in an inactive GDP-bound and an active GTP-bound conformation, and in the active GTP-bound state, Ras functions by binding and modulating the activity of a diverse array of effector proteins, such as Raf kinases, RalGEFs, and phosphatidylinositol 3-kinases[3,4]. Recently, new genes encoding the Ras-association (RA) domain have been identified, and termed the Ras-association domain family (*RASSF*) [5,6]. RASSF proteins interact either directly or indirectly with activated Ras and may serve as tumor suppressors by modulating signaling pathways associated with tumor cell growth [7]. Among 8 *RASSF *genes identified, *RASSF1 *was the most well-characterized tumor suppressor, which exhibits a frequent epigenetic inactivation in various types of human neoplasms, including lung and breast cancers [6-11]. *RASSF1 *encodes several isoforms (*RASSF1A-C)*, which are derived from alternative mRNA splicing and promoter usage. Transcriptional silencing of *RASSF1A *was observed in a considerable proportion of lung, breast, ovarian, and nasopharyngeal cancers by *de novo *methylation at the CpG islands in the promoter [8-11]. In small cell lung cancers, allelic loss of 3p21.3 was associated with *RASSF1A *methylation, suggesting that both genetic and epigenetic mechanisms are implicated in *RASSF1A *inactivation in some tumor types [9]. In addition, *RASSF1A *was identified to suppress the growth of tumor cells in both *in vitro *and *in vivo*, and Ras regulates pro-apoptotic pathway through its interaction with RASSF1A [5,8,12].

*NORE1 (RASSF5)*, a human homologue of the mouse Ras effector *Nore1*, is newly identified member of the RASSF family [13,14]. The *NORE1 *gene is located on chromosome 1q32.1, and two major transcripts (*NORE1A *and *NORE1B*) are derived from different promoter usage [14]. *NORE1A *is epigenetically inactivated by promoter hypermethylation in various cancer cell lines and primary tumors, including lung, breast, and kidney tumors [15,16]. It was also found that NORE1A mediates Ras-dependent apoptosis, and its reintroduction in defective cell lines impairs tumor cell growth in soft agar and suppresses colony formation [12,17-19]. Interestingly, it was reported that the proapoptotic effect of RASSF1 may require heterodimerization with NORE1A [20]. Very recently, it was identified that NORE1A activates the cyclin-dependent kinase inhibitor p21^WAF1 ^via promoting p53 nuclear localization, and loss of NORE1A expression correlates with loss of p21^WAF1 ^in human hepatocellular carcinoma [21]. All of these studies suggest that NORE1A might be a putative tumor suppressor in human cancers.

In the present study, we investigated the expression and mutation status of two *NORE1 *isoforms, *NORE1A *and *NORE1B *in a series of primary colorectal carcinoma tissues and cancer cell lines to explore its candidacy as a tumor suppressor in colorectal tumorigenesis. Our data demonstrate that expression of *NORE1A *and *NORE1B *is abnormally down-regulated in a substantial fraction of colorectal cancer cell lines and primary carcinomas by aberrant promoter hypermethylation. Furthermore, altered expression of *NORE1 *correlated with tumor stage and grade. We also found that both NORE1A and NORE1B exert growth suppression effect via inhibition of cellular growth and colony formation of tumor cells and enhancement of cellular response to apoptotic stress. Together, our data suggest that inactivation of *NORE1 *may play a critical role in the malignant progression of colorectal tumors.

## Methods

### Human colorectal cancer cell lines and tissue specimens

Ten human colon cancer cell lines (Caco2, Colo320, HCT116, SW403, SW620, DLD-1, WiDr, SNU-C1, SNU-C4, and SNU-C5) were obtained from American Type Culture Collection (Rockville, MD, USA) and Korea Cell Line Bank (Seoul National University, Seoul, Korea). Cell lines were maintained at 37°C in RPMI 1640 or DMEM medium supplemented with 10% fetal bovine serum (FBS) (GIBCO BRL, Carlsbad, CA, USA). A total of 80 primary colorectal tumor specimens and their adjacent normal tissues were obtained by surgical resection in the Kyung Hee University Medical Center (Seoul, Korea). Tissue specimens were snap-frozen immediately in liquid N_2 _and stored at -80°C until used. Signed informed consent was obtained from each patient. Bits of primary tumors and adjacent portions of each tumor were fixed and used for hematoxylin and eosin staining for histopathological evaluation. Tumor specimens composed of at least 70% carcinoma cells and adjacent tissues found not to contain tumor cells were chosen for molecular analysis.

### Semiquantitative reverse transcription (RT)- and DNA-polymerase chain reaction (PCR) analysis

Total cellular RNA was extracted from tissues and cell lines by standard method. One μg of DNase1-treated RNA was converted to cDNA by reverse transcription using random hexamer primers and MoMuLV reverse transcriptase (Life Technologies, Inc., Gaithersburg, MD, USA). PCR was initially performed over a range of cycles (24, 26, 28, 30, 32, 34, 36, and 38 cycles) and 2 μl of 1:4 diluted cDNA (12.5 ng/50 μl PCR reaction) undergoing 28-36 cycles was observed to be within the logarithmic phase of amplification with primers NORE1-10 (sense: 5'-CAGTTGGACTGCAGTCAGCA-3') and NORE1-9 (antisense: 5'-GGAGAGTTTCTGGAAGAGCA-3') for *NORE1A*, NORE1-5 (sense: 5-CATGAGCAGTGGGTACTGCA-3') and NORE1-9 for *NORE1B*, and G2 (sense; 5'- CATGTGGGCCATGAGGTCCACCAC-3') and G3 (antisense; 5'-AACCATGAGAAGTATGACAACAGC-3') for an endogenous expression standard gene *GAPDH*. PCR primers for *NORE1A *and *NORE1B *were designed to amplify the transcript regions of exons 2α-5 and 2β-5, respectively. PCR was done for 34 cycles at 95°C (1 min), 58-62°C (0.5 min), and 72°C (1 min) in 1.5 mM MgCl_2_-containing reaction buffer (PCR buffer II, Perkin Elmer). For genomic PCR analysis, 200 ng of genomic DNA, which was extracted from the same cells by dialysis of the DNA phase after RNA was extracted, were used for amplification of the exon 2 region of *NORE1 *with intron-specific primers G-RSF5-S (sense; 5'-GCGTGCGGGGAGACCGCAGTCT-3') and G-RSF5-AS (antisense; 5'-CTCCCAAGAACTCACAACAAA-3'). Ten μl of PCR products were resolved on 2% agarose gels. Quantitation was achieved by densitometric scanning of the ethidium bromide-stained gels. Absolute area integrations of the curves representing each specimen were then compared after adjustment for *GAPDH *level. Integration and analysis was performed using Molecular Analyst software program (Bio-Rad, Hercules, CA, USA). Semi-quantitative PCR was repeated at least three times for each specimen and mean level was calculated.

### Immunoblotting analysis

Cells were lysed in a lysis buffer containing 20 mM Tris (pH 7.4), 150 mM NaCl, 1% NP-40, 0.5% sodium deoxycholate, 0.1% sodium dodecyl sulfate (SDS), 50 mM sodium fluoride, 2 mM sodium pyrophosphate, 1 mM sodium orthovanadate, protease inhibitor cocktail, and 1 mM PMSF. The cell lysate was clarified by centrifugation and 20 to 50 μg of total protein was supplemented with Laemmli buffer and loaded on an 8% SDS-polyacrylamide gel for electrophoresis. Immunoblotting analyses were performed using antibodies specific for NORE1A (Santa Cruz Biotechnology, Santa Cruz, CA, USA) and Actin (Santa Cruz Biotechnology). Antibody binding was detected by enhanced chemiluminescence (Santa Cruz Biotechnology) using a secondary antibody conjugated to horseradish peroxidase. For reprobing with other antibodies, the membranes were incubated in a stripping buffer (0.2M glycine pH 2.2, 0.1% SDS, 1% Tween-20) at 50°C for 60 min.

### Nonisotopic PCR-single strand conformation polymorphism (SSCP) analysis

To detect possible sequence alterations in *NORE1*, we performed nonisotopic PCR-SSCP analysis. The *NORE1 *gene was amplified with 7 sets of primers that were designed to cover the entire coding region of the gene. The PCR products of over 300 bp in lengths were digested with endonuclease(s) to increase the sensitivity of SSCP analysis. Twenty μl of PCR products were mixed with 10 μl of 0.5 N NaOH, 10 mM EDTA, and 15 μl of denaturing loading buffer (95% formamide, 20 mM EDTA, 0.05% bromophenol blue, and 0.05% xylene cyanol). After heating at 95°C for 5 min, samples were loaded in wells pre-cooled to 4°C and run using 9% nondenaturating acrylamide gels containing 10% glycerol at 4-8°C and 18-22°C.

### 5-Aza-2'-deoxycytidine (5-Aza-dC) and TSA (Trichostantin A) treatment

To assess reactivation of *NORE1 *expression, cell lines showing no or low levels of *NORE1A *and/or *NORE1B *transcripts were plated in six-well tissue plates 48 h before treatment. 5-Aza-dC (Sigma, St. Louis, MO, USA) was added to the fresh medium at concentration of 1, 5, and 10 μM in duplicate and cells were harvested after 5 days. To evaluate effect of TSA on *NORE1 *expression, cells were incubated with 5-Aza-dC for 48 h and then treated with TSA (Sigma) at concentration of 250 ng/ml for 48 h.

### Bisulfite DNA sequencing analysis

Fifty ng of bisulfite-modified DNA was subjected to PCR amplification of the *NORE1A *promoter region using primers MS-NORE3 (5'-AAAGAGGCAGGGCTGAAGGACCTAGG-3') and MS-NORE6 (antisense; 5'- CCGATGGCCGGGGACGCCATGGCC-3'). The PCR products were cloned into pCRII vectors (Invitrogen, Carlsbad, CA, USA) and 5 clones of each specimen were sequenced by automated fluorescence-based DNA sequencing to determine the methylation status.

### Construction of expression plasmids, siRNA and transfection

Expression vectors encoding wild-type NORE1A and NORE1B were constructed by a PCR based approach using primers NORE1A-S (sense; 5'-ATGGCCATGGCGTCCCCGGC-3') and NORE1-AS (antisense; 5'-CCCAGGTTTGCCCTGGGATT-3') for NORE1A and NORE1B-S (sense; 5'-ATGACCGTGGACAGCAGCAC-3') and NORE1-AS for NORE1B. The PCR products were cloned into pEF6/V5-His-TOPO expression vector (Invitrogen, Carlsbad, CA, USA). Transfection of expression plasmids was performed using Effectene (QIAGEN, Hilden, Germany) according to the instruction of manufacturer. Briefly, 1 × 10^5 ^cells were transfected with plasmids-Effectene mixture for 4 h. The transfected cells were incubated with RPMI1640 medium with 10% serum for indicated duration. Detectable toxicity and apoptosis by reagent or vectors was rare. Each transfection experiment was carried out in triplicate. The transfection efficiency was monitored using a CAT assay (Roche, Mannheim, Germany) according to the instruction of manufacturer. Small interfering RNA (siRNA) duplex against NORE1A (5'-GCGCUGCACUAACUGUAAA-3'), NORE1B (5'-GCGCAGAGCAAACAUCUUU-3') and control siRNA duplex which served as negative control were synthesized by Invitrogen (Carlsbad, CA, USA). For transfection, 1 × 10^5 ^cells were plated on 60-mm-diameter dishes 24 h and incubated with a siRNA-Oligofectamine mixture at 37°C for 4 h. Fresh medium containing 1% fetal bovine serum was added and incubated for 20 h.

### Cell proliferation and apoptosis analysis

Cells were seeded in 6-well plate at the density of 1 × 10^5 ^cells per well in duplicate and were maintained in the presence of 10% FBS. The following day, cells were transfected with expression vector or siRNA as described above. Cell numbers were counted using a hemocytometer for 4 days at 24 h intervals. For flow cytometry analysis, cells were harvested 48 h after transfection and fixed with 70% ethanol and resuspended in 1 ml of PBS containing 50 mg/ml RNase and 50 mg/ml propidium iodide (Sigma, St. Louis, MO, USA). For apoptosis analysis, the cells were treated with 25 μM of etoposide for 24 h and fraction of apoptotic sub G1 phase was determined using a FACScan flow cytometry and Modfit software (Becton Dickinson, San Jose, CA, USA).

### Colony formation assay

Caco-2 cells (1 × 10^5 ^) were transfected with 2 μg of expression vectors encoding WT-NORE1A, WT-NORE1B or empty vector (pcDNA3.1). HCT116 cells were transfected with 20 pM of siNORE1A, siNORE1B or siControl. The transfected cells were maintained in the presence of G418 (400 ng) for 7 days. Colonies were fixed with methanol for 15 min and stained with 0.05% crystal violet in 20% ethanol.

### Statistical analysis

A student's *t*-test was used to determine the statistical significance of the difference. The Chi-square test was used to determine the statistical significance of expression and methylation levels between tumor and normal tissues. A *P *value of less than 0.05 was considered significant.

## Results

### Frequent reduction of NORE1 expression in primary carcinoma tissues and cancer cell lines

**Figure 1:**
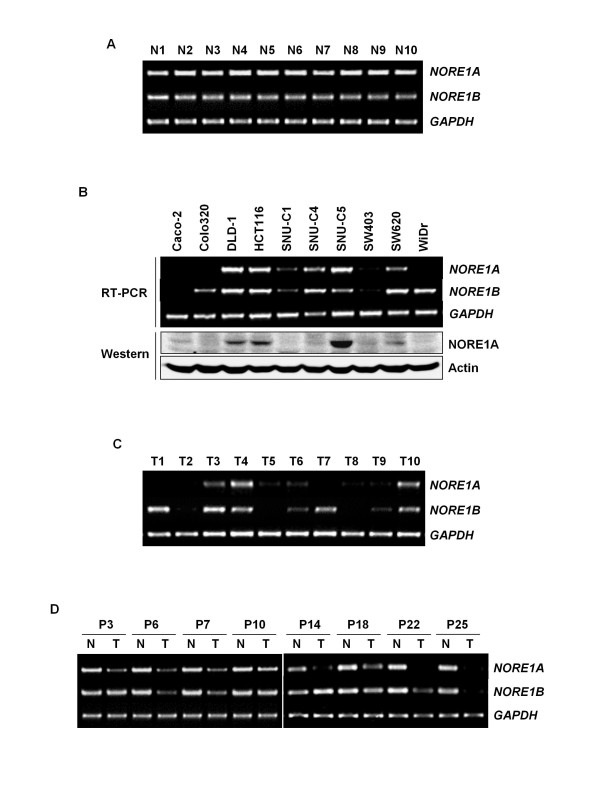
**Expression status of NORE1A and NORE1B in human colorectal tissues and cancer cell lines**. Expression of *NORE1A *and *NORE1B *mRNA in normal colorectal epithelial tissues (A). Semi-quantitative RT-PCR was performed using exon-specific primers and 10 μl of the PCR products were resolved on a 2% agarose gel. *GAPDH *was used as an endogenous control. Frequent reduction of NORE1A and NORE1B expression in colorectal cancer cell lines (B). An immunoblting assay was performed using NORE1A-specific antibody and chemiluminescence detection. Expression of *NORE1A *and *NORE1B *in primary colorectal carcinoma tissues (C). Tumor-specific reduction of *NORE1A *and *NORE1B *expression (D). *NORE1 *expression in tumor (T) and adjacent noncancerous (N) tissues were compared using matched tissue sets obtained from the same patients (P).

To explore whether *NORE1 *alteration is implicated in colorectal tumorigenesis, we initially determined expression levels of *NORE1 *transcripts in 80 noncancerous tissues and 10 cancer cell lines using semi-quantitative RT-PCR analysis. Expression of *NORE1A *and *NORE1B *transcripts was easily detectable in all noncancerous tissues tested and its levels showed no significant variations among the specimens (Figure 1A). By contrast, variable levels of *NORE1 *transcripts were observed in cancer cell lines (Figure 1B). Compared to noncancerous tissues, 70% (7 of 10) and 40% (4 of 10) of cell lines exhibited substantial reduction of *NORE1A *and *NORE1B*, respectively. Four cell lines (Caco-2, Colo320, SNU-C1 and SW403) showed low expression of both transcripts. Except WiDr, which has a *NORE1A*-specific reduction, cancer cell lines showed similar pattern in expression of *NORE1A *and *NORE1B*, suggesting that mRNA expression of *NORE1A *and *NORE1B *might be similarly controlled or affected in colorectal cancer cells. *NORE1A *and/or *NORE1B *transcripts in Caco-2, Colo320 and WiDr were detected only by nested PCR, indicating that these cancer cells expressed extremely low levels of the transcripts. An immunoblot assay using anti-NORE1A antibody revealed that protein levels of NORE1A are well consistent with its mRNA levels (Figure 1B). Together, these results demonstrate that both *NORE1A *and *NORE1B *expression is frequently down-regulated in colorectal cancer cells at the transcript level, and *NORE1A *is more commonly down-regulated compared to *NORE1B*. Likely cancer cell lines, primary tumors expressed variable levels of *NORE1A *and *NORE1B*, and a substantial fraction of tumors showed a marked reduction in expression of both transcripts (Figure 1C). In addition, among 80 matched tissue sets we examined, 46 (57.6%) and 38 (47.5%) cases revealed tumor-specific reduction (>40% reduction compared to corresponding normal tissues) of *NORE1A *and *NORE1B*, respectively while none of matched sets displayed tumor-specific elevation of the transcripts (Figure 1D). While 6 of 80 (7.5%) tumors showed *NORE1A*-specific reduction, none of primary tumors exhibited *NORE1B*-specific reduction. Except these 6 cases, expression status of *NORE1A *and *NORE1B *was fairly similar in the same tumors. These results indicate that down-regulation of *NORE1 *expression is a tumor-specific phenomenon and *NORE1A *might be a more common target for inactivation in colorectal tumorigenesis.

### Association of NORE1 reduction with tumor progression

**Figure 2:**
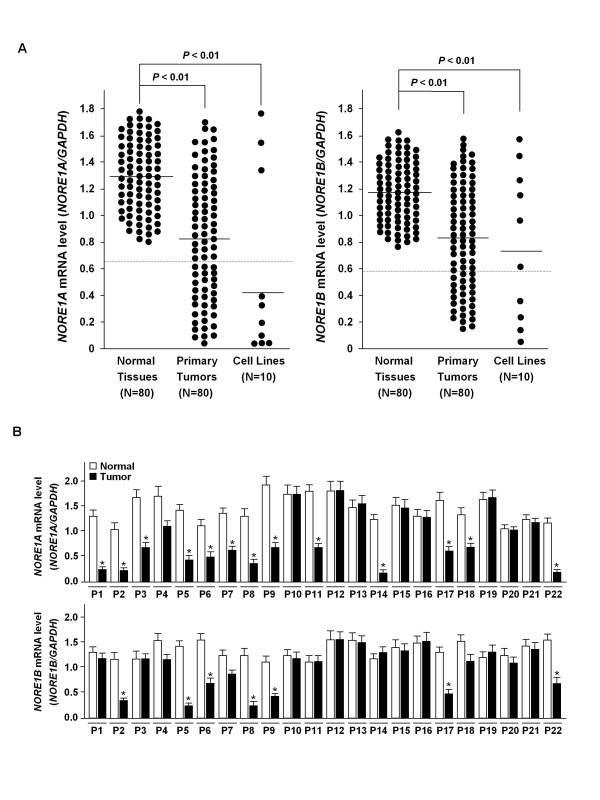
**Quantitative analysis of *NORE1 *mRNA levels in colorectal cancer cell lines and tissue specimens**. Expression levels of *NORE1A *and *NORE1B *in colorectal tissues and cell lines (A). Quantitation was achieved by densitometric scanning of RT-PCR products in ethidium bromide-stained gels. Absolute area integrations of the curves representing each specimen were compared after adjustment for *GAPDH*. Data represent means of triplicate assays (Bars, SD) Bar indicates the mean expression level of each specimen group. Expression status of *NORE1A *and *NORE1B *in matched tumor sets (B). Expression levels of *NORE1A *and *NORE1B *were compared between cancerous and adjacent noncancerous tissues from the same patients. Semi-quantitative RT-PCR was repeated at least three times for each specimen (*, P < 0.05).

**Figure 3:**
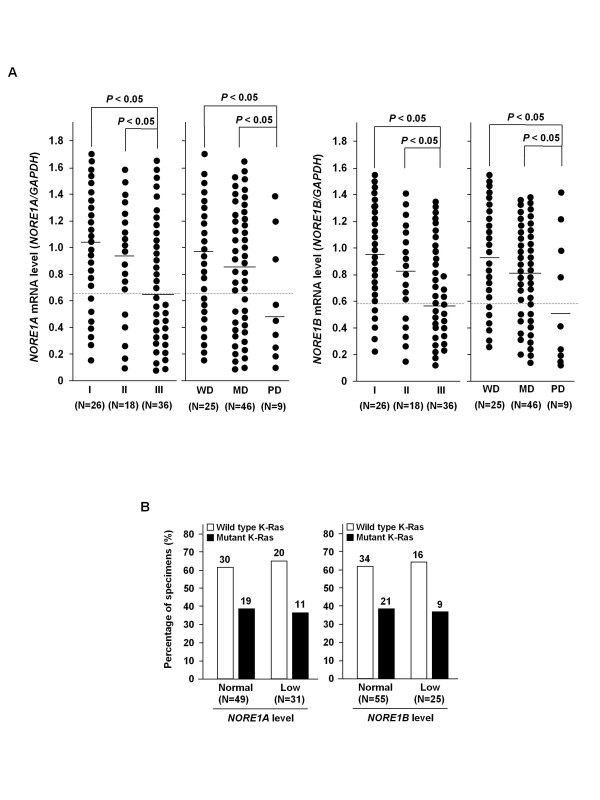
**Association of *NORE1 *alteration with tumor progression**. Correlation of *NORE1 *reduction with tumor stage and grade (A). Expression levels of *NORE1A *and *NORE1B *were compared between stages I, II, and III tumors, and between well differentiated (WD), moderately differentiated (MD), and poorly differentiated (PD) tumors. No association of *NORE1A *and *NORE1B *expression with K-Ras mutation in primary colorectal tumors (B). The mutational status of K-Ras in 80 primary carcinoma tissues was determined by SSCP and DNA sequencing analysis.

To explore the significance of *NORE1 *reduction in cancers, its expression levels were compared between noncancerous tissues, primary carcinoma tissues, and cancer cell lines. Semi-quantitative PCR was repeated at least three times for each specimen and mean levels were obtained. As shown in Figure 2A, mean of expression levels in normal tissues, primary cancers, and cancer cell lines, was determined as 1.28, 0.82, and 0.41 for *NORE1A *and 1.18, 0.83, and 0.71 for *NORE1B*, respectively. Expression levels of both transcripts were significantly low in primary cancers and cancer cell lines compared to that of normal tissues (*P *< 0.01). We arbitrarily set a value less than a half of normal mean (0.64 for *NORE1A *and 0.59 for *NORE1B*) as abnormally low level. Based on this criteria, 31 (38.8%) and 25 (31.3%) of 80 primary carcinoma tissues and 7 (70%) and 4 (40%) of 10 cancer cell lines were classified as abnormally low expressors of *NORE1A *and *NORE1B*, respectively (Figure 2A). Likely cancer cell lines, all of low *NORE1B *tumors (25 of 25, 100%) were identified to have low *NORE1A *level, showing that *NORE1A *reduction is a more common than *NORE1B *reduction in colorectal cancers (Figure 2B). Furthermore, *NORE1A *reduction was significantly high in stage III tumor (19 of 36, 52.8%) compared with stage I and II tumors (7 of 26, 26.9% and 5 of 18, 27.8%, respectively) (*P *< 0.05) and more frequent in poorly differentiated tumors (6 of 9, 66.7%) than well and moderately differentiated tumors (9 of 25, 36% and 16 of 46, 34.8%, respectively) (*P *< 0.05) (Figure 3A). Likewise, *NORE1B *reduction was significantly frequent in stage III tumors (15 of 36, 41.7%) compared with stage I and II tumors (5 of 26, 19.2% and 5 of 18, 27.8% and) (*P *< 0.05) and more common in poorly differentiated tumors (6 of 9, 66.7%) than well and moderately differentiated tumors (6 of 25, 24.0% and 13 of 46, 28.3%, respectively) (*P *< 0.05). However, *NORE1 *expression showed no association with age and gender of the patients (data not shown). Based on a previous report demonstrating that NORE1A mediates Ras-dependent apoptosis [12], we evaluated the possible association of *NORE1 *reduction with the mutational status of K-Ras in primary carcinoma tissues. The presence of K-Ras mutation was determined by SSCP and subsequent DNA sequencing analysis of K-Ras exonic regions comprising of codons 12, 13, and 61. K-Ras mutations were found in 30 of 80 (37.5%) primary tumors, but showed no significant correlation with *NORE1 *expression (Figure 3B). Together, these results indicate that abnormal reduction of *NORE1 *expression is associated with the malignant progression of colorectal tumors.

### Re-activation of NORE1 expression by 5-Aza-dC treatment

**Figure 4:**
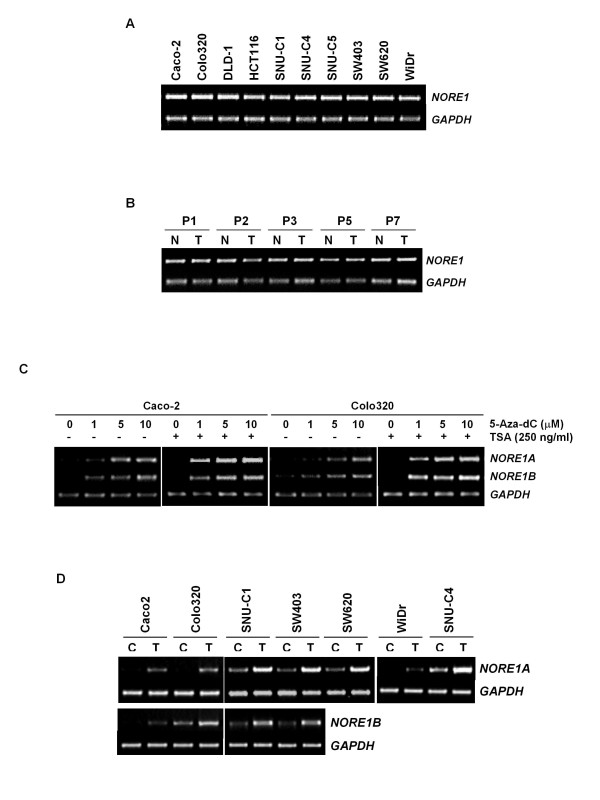
**Genomic status of *NORE1 *and re-activation by 5-Aza-dC**. Genomic level of *NORE1 *in colorectal cancer cell lines (A). The *NORE1 *gene was amplified by genomic PCR using intron-specific primer sets. Ten μl of the PCR products were resolved on a 2% agarose gel. *GAPDH *was used as an endogenous control. Comparison of *NORE1 *gene level between tumor and adjacent noncancerous tissues (B). Effect of 5-Aza-dC and TSA on *NORE1A *and *NORE1B *mRNA expression (C). Caco-2 and Colo320 cells were treated with increasing doses of 5-Aza-dC for 4 days. For combined treatment with TSA, the cells were incubated with 5-Aza-dC for 48 h and then exposed to TSA (250 ng/ml) for 48 h. Elevation of *NORE1A *and *NORE1B *mRNA expression in low expressor cell lines following 5-Aza-dC treatment (D). Cancer cell lines with low *NORE1 *level were treated with 5-Aza-dC (5 μM) for 5 days and *NORE1A *and *NORE1B *expression was evaluated by RT-PCR analysis. C, untreated control; T, 5-Aza-dC treated.

To define whether *NORE1 *reduction in cancers was caused by loss of the gene itself, genomic level of *NORE1 *was examined using quantitative genomic PCR in 10 cancer cell lines. However, none of the cell lines showed detectable reduction of *NORE1 *gene level (Figure 4A). Likewise, no difference in *NORE1 *gene level was recognized between cancer and adjacent noncancerous tissues from 80 matched tissue sets (Figure 4B). To examine the mutational status of the gene, 10 cancer cell lines and 40 primary carcinoma tissues were subjected to PCR-SSCP analysis using 7 primer sets which cover the entire coding region of *NORE1*. However, we failed to find any types of mutation leading to amino acid substitution (data not shown). These observations indicate that genomic deletion and mutation of *NORE1 *is not common and may not be associated with abnormal reduction of *NORE1 *expression in colorectal cancers. Next we tested whether *NORE1 *down-regulation is associated with by DNA hypermethylation. Cancer cell lines with low *NORE1 *level were treated with the demethylating agent 5-Aza-dC and the histone deacetylation inhibitor Trichostantin A and its effect on transcript expression was determined by RT-PCR analysis. Our initial test using two cell lines (Caco-2 and Colo320) demonstrated that expression of *NORE1A *and *NORE1B *is re-activated by 5-Aza-dC treatment in a dose-associated manner and its levels are further elevated in the presence of TSA (Figure 4C). As shown in Figure 4D, we observed that transcript levels of *NORE1A *and *NORE1B *are markedly increased in all low expressor cell lines following 5-Aza-dC treatment, indicating that *NORE1 *expression is down-regulated in these cancer cells at the transcription level by abnormal DNA hypermethylation.

### Aberrant promoter CpG sites hypermethyation of NORE1A in colorectal cancers

**Figure 5:**
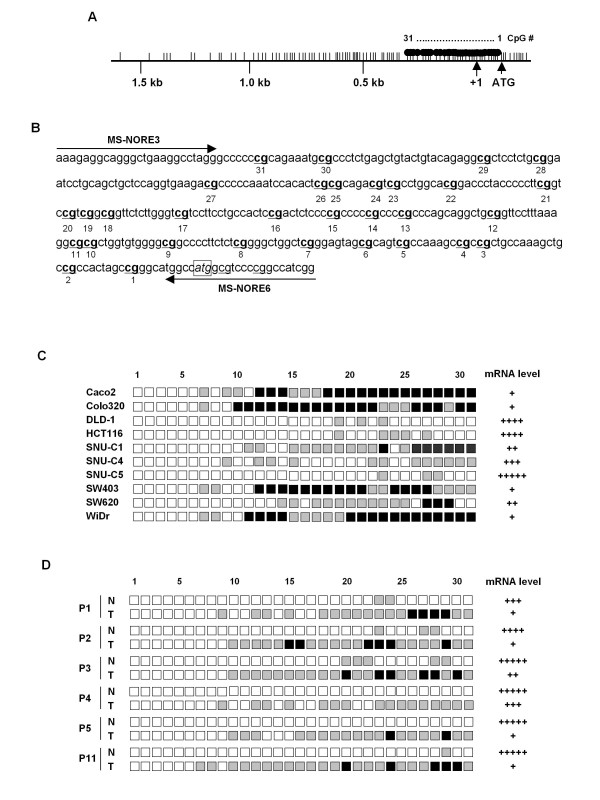
**Methylation status of CpG sites in the *NORE1A *promoter**. A map of the CpG sites within the proximal region of the *NORE1A *promoter (A). CpGs sites are represented by vertical lines. The transcription start site is indicated by an arrow at +1. Nucleotide sequences of *NORE1A *promoter region examined by bisulfite DNA sequencing analysis (B). Thirty one CpG sites (bold) examined are numbered 1-31 and primers for PCR were indicated. The ATG start codon is indicated (boxed). Methylation status of 31 CpG sites in the *NORE1A *promoter and its association with mRNA level in colorectal cancer cell lines (C). The promoter region comprised of 31 CpGs was amplified by PCR. The PCR products were cloned and 5 plasmid clones were sequenced for each cell line. Percent methylation was determined by the number of alleles containing a methylated CpG at each position. Black, gray, and white squares represent complete methylation (4-5 clones), partial methylation (1-3 clones), and unmethylation (0 clone), respectively. Comparison of CpG sites methylation between primary tumors and adjacent noncancerous tissues (D). N, noncancerous tissue; T, tumor tissue; P, patient.

To elicit whether tumor-specific reduction of *NORE1 *expression is caused by the aberrant promoter hypermethylation, the methylation status of 31 CpG sites located within the proximal region of the *NORE1A *promoter was characterized (Figure 5A and 5B). Bisulfite DNA sequencing was performed for 10 cell lines and 6 matched tissue sets showing tumor-specific reduction of *NORE1A *transcript levels. Five PCR clones from each specimen were randomly selected to determine methylation frequency at individual CpG sites (complete methylation; 4-5 clones, partial methylation; 1-3 clones, unmethylation; 0 clone). As summarized in Figure 5C, a strong correlation between methylation frequency and transcript level was found in cancer cell lines. Interestingly, the majority of the 21 CpG sites (CpG site numbers 11-31) was completely or partially methylated in cancer cell lines showing low level of expression (Caco2, Colo320, SNU-C1, SW403, SW620, and WiDr), while only 3-5 (9.7-16.1%) sites were partially methylated in cell lines showing strong expression (DLD-1 and HCT116). SNU-C4 cells, which express an intermediate level of *NORE1A *transcript, showed only partial methylation at 12 CpG sites. Likewise, 6 primary tumors with low transcript level showed methylation at 16-24 (51.6-77.4%) sites, whereas methylation was very rare (0-3 sites) in its adjacent noncancerous tissues (Figure 5D). Therefore, these results clearly demonstrate that aberrant promoter CpG sites hypermethylation is tightly associated with decreased expression of *NORE1 *transcript in colorectal cancers.

### Inhibition of tumor cell growth by NORE1A and NORE1B

**Figure 6:**
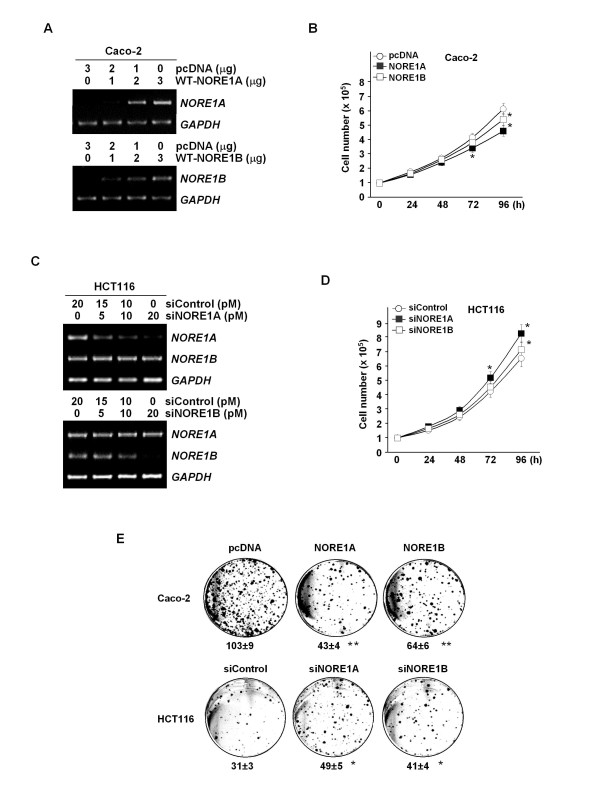
**Suppression of tumor cell growth by NORE1A and NORE1B**. Transient transfection of expression vectors encoding WT-NORE1A and WT-NORE1B in Caco-2 cells (A). A dose-associated expression of NORE1A and NORE1B was determined at 72 h post-transfection using semi-quantitative RT-PCR. Suppression of Caco-2 cell growth by NORE1A or NORE1B (B). Caco-2 cells were transfected with 2 μg of WT-NORE1A or WT-NORE1B and cell numbers were counted at 24 h intervals. The assay was performed in triplicate (*, P < 0.05). SiRNA-mediated knockdown of NORE1 expression in HCT116 cells (C). The cells were transfected with increasing doses of siRNA and NORE1A and NORE1B levels were determined by RT-PCR at 72 h post-transfection. Anti-proliferative role of endogenous NORE1A and NORE1B (D). HCT116 cells were transfected with 50 nM of siNORE1A, siNORE1B or siControl and cell numbers were counted at 24 h intervals. The assay was performed in triplicate (*, P < 0.05). Suppression of colony formation of tumor cells by NORE1 (E). Caco-2 and HCT116 cells transfected with 2 μg of expression vectors or siRNA were maintained in the presence of G418 (400 ng) for 7 days. The assay was performed in triplicate (*, P < 0.05; **, P < 0.01).

**Figure 7:**
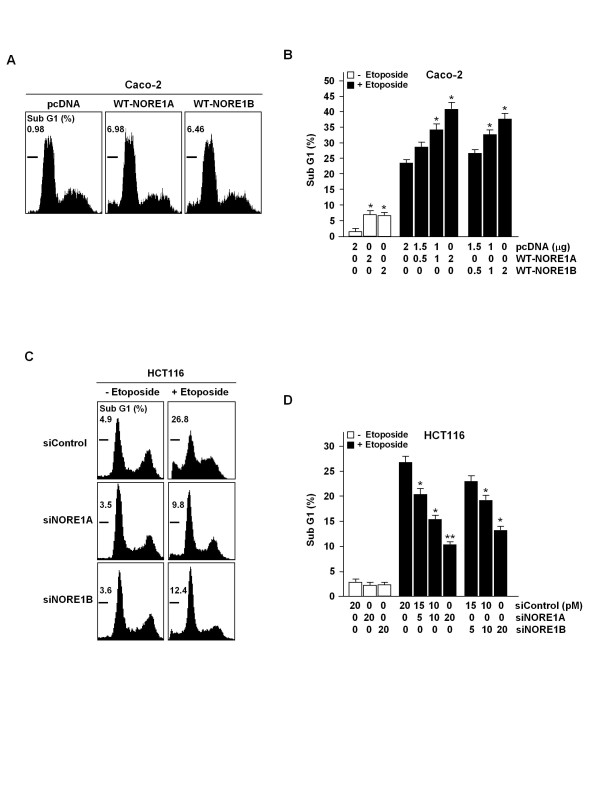
**Anti-apoptotic function of NORE1A and NORE1B**. Effect of NORE1 expression on baseline and etoposide-induced apoptosis (A, B). Caco-2 cells were transfected with WT-NORE1A or WT-NORE1B and percentage of sub G1 phase was determined by flow cytometric analysis. Caco-2 cells transfected with WT-NORE1 were treated with etoposide (25 μM, 24 h). Effect of NORE1 knockdown on etoposide-induced apoptosis (C, D). HCT116 cells transfected with siNORE1 were treated with etoposide (25 μM, 24 h).

To define the roles for NORE1 in tumor growth, we initially evaluated effects of its expression on cellular growth using ectopic expression of wild-type (WT)-NORE1A and WT-NORE1B in Caco-2 and siRNA-mediated knockdown of endogenous NORE1A and NORE1B expression in HCT116. Transfection of WT-NORE1 and siNORE1 led to a dose-associated induction and knockdown of NORE1, respectively (Figure 6A and 6C). Cell number counting assay showed that cellular growth of Caco-2 is suppressed by expression of NORE1A or NORE1B, and growth of HCT116 is facilitated by knockdown of NORE1A or NORE1B (Figure 6B and 6D). Compared to empty vector-transfected control, cellular growth of Caco-2 showed approximately 29.5% and 18.0% reduction at 4 days after transfection of WT-NORE1A and WT-NORE1B, respectively. Likewise, cellular growth of HCT116 exhibited approximately 24.6% and 7.7% increase by transfection of siNORE1A and siNORE1B, respectively. These results indicate that both NORE1A and NORE1B exert growth inhibition effect and NORE1A might have more potent anti-proliferative ability compared to NORE1B in colorectal epithelial cells. A flow cytometric analysis of the sub G1 phase revealed that expression of NORE1A and NORE1B enhances both baseline (pcDNA 0.98%, WT-NORE1A 6.98%, and WT-NORE1B 6.46%) and etoposide-induced apoptosis (pcDNA 23.2%, WT-NORE1A 40.9%, WT-NORE1B 37.4% in 2 μg transfection) (Figure 7A and 7B). Similarly, in HCT116 cells, etoposide-induced apoptosis was significantly decreased by knockdown of NORE1A or NORE1B (Figure 7C and 7D). The apoptotic response of both Caco-2 and HCT116 cells to etoposide was up- and down-regulated by restoration or knockdown of NORE1A and NORE1B in a dose-associated manner. Consistent with these, ectopic expression of NORE1A and NORE1B led to approximately 58.3% and 37.9% decrease in colony forming ability of Caco-2 cells while knockdown of NORE1A and NORE1B caused approximately 61.3% and 32.3% increase in colony forming ability of HCT116 cells, further demonstrating that both NORE1A and NORE1B have growth suppression activities (Figure 7E).

## Discussion

In the present study, we demonstrated that expression of *NORE1A *and *NORE1B *is down-regulated at the transcription level in a substantial fraction of colorectal cancer cell lines and primary carcinomas. Abnormal reduction of *NORE1A *and *NORE1B *was identified to be a tumor-specific phenomenon and correlated with advanced stage and high grade of tumors. In both established cell lines and primary tumors, downregulation of *NORE1A *expression was tightly associated with aberrant promoter CpG sites hypermethylation. Moreover, NORE1A and NORE1B were found to suppress cellular growth and colony forming ability of tumor cells and enhance tumor cell response to apoptotic stress, indicating that both NORE1 isoforms have growth suppression functions. Collectively, these results suggest that epigenetic silencing of *NORE1 *might contribute to the malignant progression of human colorectal cancers.

Chromosome 1q32 region exhibits loss of heterozygosity in many types of human cancer including colorectal cancer, thus suggested to harbor tumor suppressor genes [22,23]. *NORE1 *was predicted as a target of deletion at 1q32.1, and allelic loss of the gene was suggested as a plausible mechanism underlying its low expression in tumor cells [13]. In this study, however, genomic analysis of *NORE1 *revealed that deletion or sequence alteration of *NORE1 *is very rare in cancers, suggesting that genomic deletion of the gene might not be a main cause leading to loss or abnormal reduction of its expression in colorectal cancers.

Hypermethylation in CpG-rich region is strongly associated with transcriptional silencing, and hypermethylation at CpG sites in transcription regulatory region is a critical event leading to the epigenetic inactivation of tumor suppressor genes [24,25]. Hypermethylation of the *NORE1 *gene has been reported in several cancers, including breast, lung, kidney, and colorectal cancers [15,26]. It was also reported that epigenetic alteration of *NORE1A *is confined to lung tumors with a wild-type *K-Ras*, suggesting that epigenetic inactivation of *NORE1A *and mutational activation of K-Ras might play a cooperative role in lung tumorigenesis [27]. In the present study, we found that expression of both *NORE1A *and *NORE1B *is frequently decreased in primary colorectal carcinoma tissues as well as cancer cell lines. However, no association was identified between expression status of *NORE1A *or *NORE1B *and mutational status of *K-Ras *in 80 primary carcinomas specimens we examined. Interestingly, abnormal reduction of *NORE1A *was more frequent compared to that of *NORE1B *and none of cell lines and primary tumors showed *NORE1B*-specific reduction, suggesting that *NORE1A *might be a more critical target of inactivation in colorectal tumorigenesis. Expression of both *NORE1 *isoforms was re-activated in cell lines by treatment with 5-Aza-dC and the *NORE1A *promoter was identified to be aberrantly hypermethylated in cancer cell lines and primary tumors expressing low levels of *NORE1A *transcript. Methylation frequency of 31 CpG sites in the proximal region of *NORE1A *promoter was much higher in tumors showing low mRNA expression compared to adjacent noncancerous tissues with normal mRNA level. Moreover, the methylation levels in both cancer cell lines and primary tumors showed a tight correlation with mRNA expression status. In particular, aberrant methylation of 21 CpG sites (numbered 11-31, Figure 5B) was most tightly associated with gene silencing, indicating that CpG dinucleotides in this region may play an important role for the regulation of *NORE1A *transcription. Therefore, our data support that *NORE1 *is epigenetically inactivated in a substantial fraction of colorectal cancers, and CpG sites hypermethylation of the 5' upstream region of the gene is crucial for its transcriptional silencing. It could be assumed that the hypermethylation of CpG sites in this region might block the access of cis-acting transcription factors to their binding sequences presumably by direct inhibition of binding or through the establishment of a repressed chromatin structure at the methylated CpG island [28,29].

Although NORE1 has been identified as a potential tumor suppressor, the biological function of NORE1 and the significance of its inactivation during tumorigenesis have not been well understood. Previous studies showed that NORE1A mediates Ras-dependent apoptosis and suppresses tumor cell growth and colony formation, and the proapoptotic effect of RASSF1 requires the heterodimerization with NORE1A [12,17-20]. In the present study we demonstrated that cellular growth and colony forming capability of colorectal cancer cells are significantly suppressed and their responses to apoptotic stress are markedly increased by expression of either NORE1A or NORE1B, indicating that both NORE1A and NORE1B have growth suppression abilities. Transient expression and siRNA-mediated knockdown assays revealed that compared to NORE1B, NORE1A evokes more potent growth inhibition effects in two different cells. Although further molecular analyses are required, this finding might correlate with our expression data supporting that *NORE1A *is a more critical target of inactivation in colorectal tumorigenesis. Interestingly, a recent study demonstrated that NORE1A activates the cyclin-dependent kinase inhibitor p21^WAF1 ^via promoting p53 nuclear localization, and loss of NORE1A expression correlates with tightly with loss of p21^WAF1 ^in human hepatocellular carcinoma [21]. Considering that p21^WAF1 ^is a crucial target of p53-mediated cell cycle arrest, this finding raises the interesting possibility that NORE1A could be a potential component, which plays an important role in functional connection between Ras and p53 signaling [21]. Although we did not evaluate the possible role of NORE1 in p53 signaling, our finding of the significant growth suppression of mutant p53-carrying Caco-2 cells by NORE1A or NORE1B indicates that both NORE1A and NORE1B have p53-independent growth arrest and apoptosis-enhancing functions. It has been well known that oncogenic activation of Ras and mutational inactivation of p53 play a critical role in colorectal tumorigenesis. In this context, expression status of NORE1 and its association with activation status of Ras and p53 could provide valuable information for the molecular mechanisms underlying colorectal tumor progression. In the present study, we observed that reduced expression of *NORE1 *is associated with histopathological characteristics of tumors, such as tumor stage and grade. However, no association was recognized between expression of *NORE1A *or *NORE1B *and mutation of *K-Ras*. In contrast to cancerous tissues, abnormal reduction or promoter hypermethylation of *NORE1 *was not found in corresponding nonneoplastic mucosa. Based on previous reports that DNA methylation occurs early in the multistep process of carcinogenesis [30-32], our data raises the possibility that epigenetic inactivation of *NORE1 *might be implicated in the development and/or early progression of colorectal tumors.

## Conclusions

Both *NORE1 *and *NORE1B *is significantly down-regulated in a considerable fraction of colorectal cell lines and primary tumors by aberrant DNA hypermethylation, and its abnormal reduction correlates with advanced stage and higher grade of tumor. Restored expression of NORE1A or NORE1B decreases cellular growth and colony forming ability of colorectal tumor cells and enhances cellular response to apoptotic stress. These findings thus suggest that epigenetic inactivation of *NORE1 *might play a crucial role in the malignant progression of colorectal tumors, possibly by providing selective growth advantage for colorectal epithelial tumor cells.

## Competing interests

The authors declare that they have no competing interests.

## Authors' contributions

CKL, JHL, TKH and MJK carried out the expression studies and statistical analysis and drafted the manuscript. BKR, YH, JJS and JYJ carried out methylation studies. MGL and SIJ carried out the immunoblot, cell growth, apoptosis, and colony formation assays. KYL participated in the design of the study. HJK and SGC conceived of the study, and participated in its design and coordination. All authors read and approved the final manuscript.

## Pre-publication history

The pre-publication history for this paper can be accessed here:

http://www.biomedcentral.com/1471-2407/10/577/prepub
